# Meta-omics reveals subgingival plaque reconstruction dynamics

**DOI:** 10.1080/20002297.2025.2569528

**Published:** 2025-10-13

**Authors:** Fangjie Zhou, Yajie Wu, Biao Ren, Yuchuan Liu, Kaihua Luo, Qinyang Li, Fangting Huang, Xian Peng, Yuqing Li, Zhifei Su, Jiyao Li

**Affiliations:** a State Key Laboratory of Oral Diseases, National Clinical Research Center for Oral Diseases, West China Hospital of Stomatology, Sichuan University, Chengdu, China; b Department of Cariology and Endodontics, West China Hospital of Stomatology, Sichuan University, Chengdu, China; c Tianfu Jiangxi Laboratory, Chengdu, China; d Shanghai OE Biotech Co., Ltd., Shanghai, China; e Department of Stomatology, Southwest Hospital, Third Military Medical University (Army Medical University), Chongqing, China; f Hospital of Stomatology, Sun Yat-sen University, Guangzhou, China

**Keywords:** Oral microbiota, subgingival plaque, dynamics, reconstruction, metagenome

## Abstract

**Background:**

The homeostasis of the subgingival microbiome is crucial for periodontal health, although the dynamics governing its community variation remain insufficiently studied. This study aims to investigate the dynamics of subgingival microbiota reassembly after disruption, focusing on core taxa, functions, and driving forces.

**Methods:**

339 subgingival plaques in periodontally healthy states were collected before and after ultrasonic cleaning across 12 timepoints for 1 year. All samples underwent full-length 16S rRNA sequencing; 30 were selected for metagenomic sequencing.

**Results:**

Our findings revealed that disturbed subgingival microbiota underwent short-term disruptions but subsequently reverted to baseline, maintaining stability within a year. Homogeneous selection dominated assembly, driving convergent structure under consistent pressure. Such a recovery process was accompanied by key taxa increased sequentially: *Pseudomonas fluorescens* early, *Haemophilus parainfluenzae* mid-stage, and *Capnocytophaga spp*. late. Functionally, reconstruction began with energy metabolism, expanded via biofilm formation and LPS biosynthesis mid-stage, and involved late apoptosis and complex amino acid metabolism. Microbial interactions, including positive regulation from *Veillonella HMT 780* to *Fusobacterium HMT 248*, internally drove community assembly.

**Conclusion:**

Our study clarifies species and functional dynamics during subgingival microbiota reconstruction and maps time-directed networks among stage-specific bacteria, offering a theoretical basis for targeted microbiome regulation.

## Graphical Abstract

**Figure:**
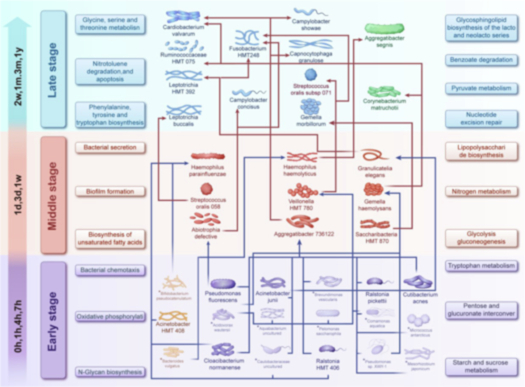

## Introduction

The stable microbial community plays an essential role in maintaining the functionality of the community and the health of host [1]. The stability of microbial communities varies among individuals and niches. For example, the gastrointestinal tract and oral microbiomes exhibit greater stability than nasal and skin microbiomes [2]. Despite perturbations such as diet and hygiene measures, a stable microbiome is maintained in a harmonious cavity. While excessive external disturbances, such as pathogen infection or the abuse of antibiotics, may lead to dysbiosis and thus induce disease [3–5]. Microbiome dynamics are pivotal indicators and potential drivers of microbial stability [6]. Understanding the dynamic characteristics of the microbiota is essential for achieving targeted microbial regulation [7]. Sequential directed interactions among core species *in situ* are a crucial aspect of microbiome dynamics. Identifying the evolutionary characteristics and dynamic factors in the assembly of healthy microbiome can help explore the core species of community dynamics and infer causal interactions among their members [8].

The oral cavity serves as a crucial interface for the exchange of substances with the external environment, and its microorganisms are susceptible to perturbations from dietary intake and hygiene measures. Normally, oral microbiomes of healthy individuals can withstand external disturbances and maintain a stable equilibrium both internally and in relation to the host [9,10]. Subgingival plaque, located in the gingival sulcus of the host, contacts the dental surface and the conjunctive epithelium in both free and adherent states. The disturbance of subgingival microbiome is a crucial initiating factor for periodontal disease [11]. Previous studies have investigated the dynamics of the subgingival microbiome, particularly its healthy-state community formation and disease-state community transition [9,12–16]. When faced with external disturbances, how healthy-state subgingival microbiome maintain or recover their structure and function remains unknown. Studying the dynamic characteristics of subgingival microbiome reconstruction in healthy individuals aids in understanding the core species and network relationships within the community and provides insights for addressing microbiome disturbances in disease states.

Longitudinal studies are the cornerstone of understanding community dynamics, encompassing the baseline dynamics of an undisturbed microbiome and its response to perturbations [17,18]. Perturbation experiments, including dietary shifts and clinical interventions, offer valuable insights into the structure and dynamics of microbial communities [19]. For example, the bowel microbiome recovers rapidly after bowel cleansing [20]. However, broad-spectrum antibiotics can cause the loss of taxa within the community, resulting in incomplete recovery of the microbial community after disturbance [21]. Current intervention approaches often suffer from excessive host interference or microbial community disruption, which complicates the identification of key microbial dynamics within complex ecological networks. To address these limitations, *in situ* longitudinal studies employing targeted microbiome perturbations are needed. Professional mechanical plaque removal via ultrasonic scaling provides an ideal model for investigating oral microbial community dynamics under controlled ecological shifts [22].

Although dental plaque research has been conducted for more than 120 years [23], high-resolution dynamic analyses of subgingival plaque reestablishment at the species level remain limited. Furthermore, the evolutionary patterns and dynamic factors governing subgingival microbial community assembly are still poorly understood. In this longitudinal 1-year study, we collected 339 subgingival plaque samples from periodontally healthy individuals following ultrasonic scaling at 12 sequential time points. Using full-length 16S rRNA and metagenomic sequencing, we characterized microbial dynamics at the species and functional levels, identified core drivers of community assembly, and inferred directed ecological interactions.

## Materials and methods

### Study design and procedures

This study received approval from the Medical Ethics Committee of West China Stomatology Hospital, Sichuan University (Protocol Number: WCHSIRB-D-2019-185), and informed consent was obtained from all participants. Based on the ‘Classification of Periodontal and Peri-Implant Diseases and Conditions’, 30 periodontally healthy subjects were recruited from visitors to West China Stomatology Hospital. Eligible participants were aged 18−30 years, with intact periodontal tissue, no clinically detectable inflammation, no clinical attachment loss or bone loss, a probing depth of ≤3 mm, and <10% bleeding on probing sites. Exclusion criteria included non-Han ethnicity, severe caries, active oral infectious diseases, systemic diseases or drug allergies, smoking, irregular tooth alignment, systemic antibiotic use within the past three months, topical antibiotic use within the past week, or periodontal treatment within the past year. Teeth affected by local factors such as fillers, orthodontic instruments, or splint devices that could influence plaque attachment were also excluded. Following ultrasonic cleaning, all subjects received oral health education and follow-up care. This included verbal and written instructions on proper brushing and flossing techniques. Participants were provided with toothpaste, toothbrushes, and dental floss to maintain oral hygiene during follow-up. They were also instructed to report any antibiotic use or dental treatments received throughout the study period.

### Sample collection and DNA extraction

Demographic information (age, gender, residence) and oral hygiene habits were collected via questionnaires before scaling. Trained investigators performed oral clinical examinations, recording the percentage of bleeding on probing sites and probing depths. Supragingival and subgingival scaling were conducted after the initial sample collection. Subgingival plaques were collected at specific intervals post-scaling: 0, 1, 4, 7 h, and 1, 3, 7, 14 days, 1, 3 months, and 1 year. Subjects were instructed to refrain from oral hygiene measures for 2 h prior to sampling. The supragingival plaque at the target site was removed, and sterile cotton rolls were used to isolate the sampling area. Subgingival plaque samples were collected from six sites (MB/La, B/La, DB/La, MP/L, P/L, DP/L) on each of six Ramfjord index teeth (#16, # 21, #24, #36, #41, #44). A sterile 35# paper point was inserted deep into the gingival sulcus at each target site, left in place for 30 s, then removed and placed into a sterile EP tube. Samples were transported under ice and frozen at −80 °C within 2 h of collection. Microbial DNA was extracted from 339 samples using the E.Z.N.A.® Soil DNA Kit (Omega Bio-Tek, Norcross, GA, USA).

### 16S rRNA gene full-length sequencing and bioinformatics analysis

The V1–V9 regions of the 16S ribosomal RNA gene were amplified by polymerase chain reaction (PCR) (98 °C for 2 min, followed by 25 cycles at 98 °C for 10 s, 55 °C for 30 s, and 72 °C for 90 s, and a final extension at 72 °C for 5 min) using primers 27F: 5'-AGRGTTYGATYMTGGCTCAG-3’ and 1492R: 5'-RGYTACCTTGTTACGACTT-3’. After the purification of the PCR products, the SMRT bell 1.0 Template Prep Kit 1.0 (PacBio, Menlo Park, CA, USA) was used to construct the sequencing library. Sequencing was then performed on the Pacbio RS II platform. The circular consensus sequencing (CCS) reads were filtered to generate high-quality sequences, which were subsequently clustered into operational taxonomic units (OTUs) at a 99% similarity threshold. These qualified sequences were then taxonomically classified using the RDP classifier through sequential alignment against the expanded Human Oral Microbiome Database (v15.23), the SILVA ribosomal RNA database (v138.2), and the GenBank database (release262). Although this multi-database approach enhances annotation sensitivity, it may also capture rare or transient taxa that are not typically regarded as part of the core oral microbiota. The Shannon index was calculated using QIIME (v.1.9.1). Principal co-ordinates analysis (PCoA) was conducted using a Weighted-UniFrac distance matrix. Additionally, the paired distance between samples at each subsequent time point and before scaling was calculated using the Weighted-Unifrac distance algorithm. The iCAMP R language package (v.1.5.12) was used to evaluate the mean nearest taxon distance (MNTD) and nearest taxon index of samples based on the community composition and phylogenetic tree of samples. βMNTD was generated by the zero model, and βNTI was calculated by the formula of standardized effect size. The proportion of ecological processes for each time point sample was calculated using the βNTI matrix, combined with Raup-Crick. Annotated species were divided into modules using the weighted gene co-expression network analysis (WGCNA, version1.71) software package (power = 12, minimum number of included species = 7, sensitivity = 4) and named by color, then correlated with time and visualized with a heat map. Phenotypic proportions were calculated using the BugBase R package based on OTU matrix and displayed with histograms.

### Shotgun metagenomic sequencing and bioinformatics analysis

Thirty subgingival plaque samples were selected by microPITA for metagenomic sequencing. The extracted DNA, after being fragmented, washed, end-repaired, spliced, and enriched, was sequenced on the Illumina NovaSeq 6000 platform. Clean data processed using Trimmomatic (v.0.36) were assembled into contigs of ≥ 500bp using MEGAHIT (v.1.1.2). Prodigal (v.2.6.3) was used to predict open reading frames (ORFs). A non-redundant gene catalog was constructed using CD-HIT (v.4.5.7) with 90% coverage and a threshold of 95% identity. DIAMOND (v.0.9.7) performed taxonomic annotation based on the NR database, and the non-redundant gene sets were mapped into the Kyoto Encyclopedia of Genes and Genomes (KEGG) database using DIAMOND (v.0.9.7) software to acquire functional information corresponding to the gene sequences. Non-metric multidimensional scaling (NMDS) of the annotated gene entries and the species abundance matrix was performed using QIIME (v.1.9.1). Significant differences in functional entries between phases were identified using linear discriminant analysis effect size (LEfSe). A Spearman correlation analysis was carried out between the relative abundance of species obtained from 16S rRNA gene full-length sequencing and the relative expression of functional entries obtained from metagenomic sequencing.

## Results

### Recovery of disturbed subgingival microorganisms to their original state

**Figure 1.**
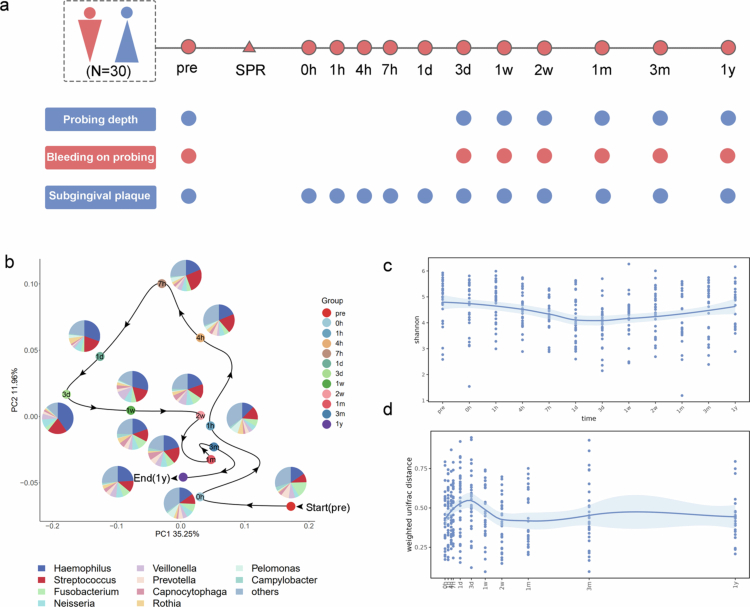
Study flow and reconfiguration of subgingival microbiota after disturbance. (a) Subgingival plaque samples were collected from 30 subjects at 12 time points, including pre-scaling and at hours 0, 1, 4, 7, days 1, 3, 7, 14, months 1, 3, and year 1 post-scaling. (b) Multidimensional analysis based on full-length 16S rRNA gene sequences from subgingival bacterial communities. The dots represent the positions of the centroids of all sampled microbiomes on the PCoA axis at each time point, connected sequentially by lines. Pie charts depict the top 10 bacteria at the class-genus level. (c) Changes in the alpha diversity of subgingival microbiota over time, with the shadow around the line indicating a 95% CI. (d) The weighted UniFrac distance between subgingival microbiota at each time point and the baseline, with the shadow around the line indicating a 95% CI.

To investigate the sequential succession characteristics of community reconstruction following scaling disturbances, subgingival plaque samples were collected from 30 subjects at 12 time points, including pre-scaling and at 0, 1, 4, 7 h, and 1, 3, 7, 14 days, 1, 3 months, and 1 year post-scaling, between 2020 and 2021 (Figure 1a). Demographics and general clinical data are summarized in Suplementary Table 1. During the follow-up period, no participants reported the use of antibiotics or any dental treatments. The bacterial communities were analyzed using full-length 16S rRNA gene sequencing on the PacBio Sequel II platform to identify the bacterial composition of the reconstruction subgingival plaque with enhanced accuracy. A total of 339 subgingival plaque samples were successfully sequenced, all reads were assigned to 210 bacterial species listed in the expanded Human Oral Microbiome Database (eHOMD), SILVA ribosomal RNA database, and GERRNGENE database with >99% identity.

The clinical data indicated that the periodontal status of the subjects remained healthy for one year following scaling (Suplementary Table 2). Microbiome profiling demonstrated the remarkable resilience of subgingival ecosystems, with both taxonomic composition and community structure showing high similarity between preoperative baselines and postoperative timepoints at 3 months and 1 year (Suplementary Figure S1), indicating robust ecological homeostasis in periodontal health. To elucidate the dynamic pattern of subgingival microbiome reconstruction, we characterized microbiota profiles by time point. Principal coordinates analysis (PCoA) visually illustrated this dynamic process. The immediate post-intervention phase (0 hour−3 day) was marked by significant community disruption, evidenced by increased *β*-diversity and maximal structural deviation from baseline at day 3 (Figure 1b). The microbiome then progressively reverted to a state closely resembling baseline levels within 1 week to 1 year, demonstrating significant temporal variability. Regular changes in bacteria such as *Capnocytophaga*, *Fusobacterium*, and *Haemophilus* marked this reconstruction. Corresponding with the PCoA trends, microbial α-diversity decreased post-cleaning, reaching its lowest point on day 3, and eventually returned to the baseline state (Figure 1c). The Weighted-Unifrac distance algorithm was applied to assess differences in paired microbiome samples between the baseline and each subsequent time point, revealing that differences peaked on day 3 post-cleaning before stabilizing (Figure 1 d). All results demonstrated a high level of concordance, confirming that disturbed subgingival microorganisms recover to their original state.

### Homogeneous selection dominates the assembly processes of disturbed subgingival microbiome reconstruction

**Figure 2.**
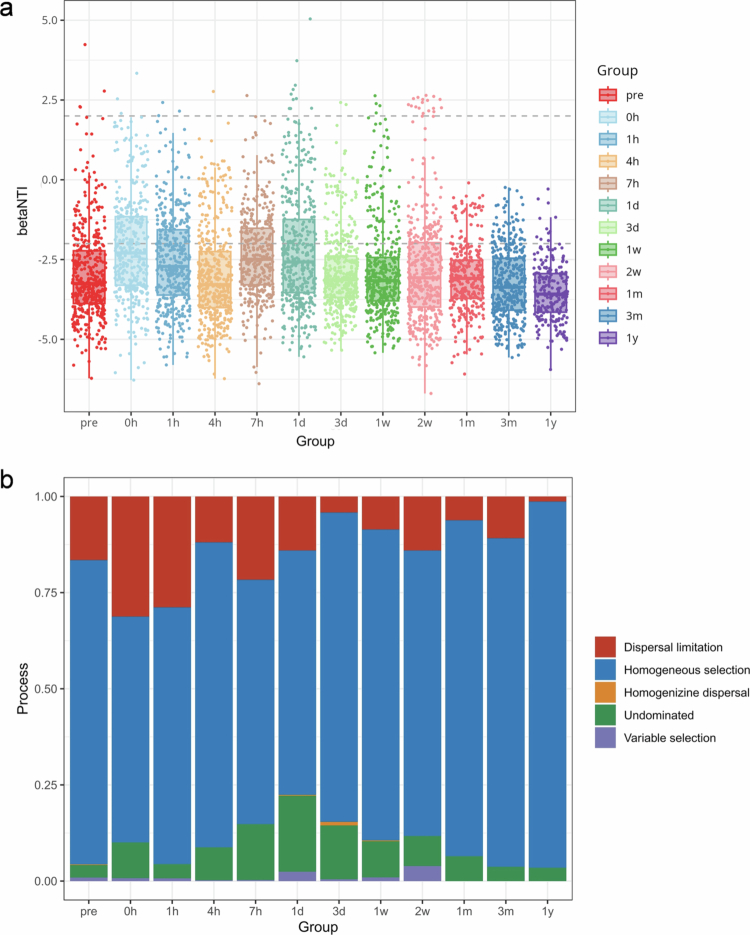
Assembly processes of subgingival microbial community reconstruction. (a) The beta nearest taxon index (βNTI) derived from null models in microbial communities. (b) The proportion of dispersal limitation, homogeneous selection, homogenizining dispersal, an undominated process, and variable selection in the subgingival microbial assembly processes.

To investigate the dominant ecological processes shaping healthy periodontal subgingival microbiome, this study calculated the *β*-nearest taxon index (βNTI) based on a null model. The average βNTI values for bacterial communities at each time point were below −2 (Figure 2a), indicating that the assembly processes were primarily driven by deterministic mechanisms. The assembly of subgingival microbial communities was governed by dispersal limitation, homogeneous selection, and non-dominant processes. Homogeneous selection emerges as the dominant driver across all time points (Figure 2b), which refers to the selection process under consistent biotic/abiotic environmental conditions that guides communities to evolve in a more similar direction. These results verified that individual communities are subject to comparable selection pressures during each stage of subgingival microbiome reestablishment.

### Reconstruction process forms distinct time-dependent bacterial modules

**Figure 3.**
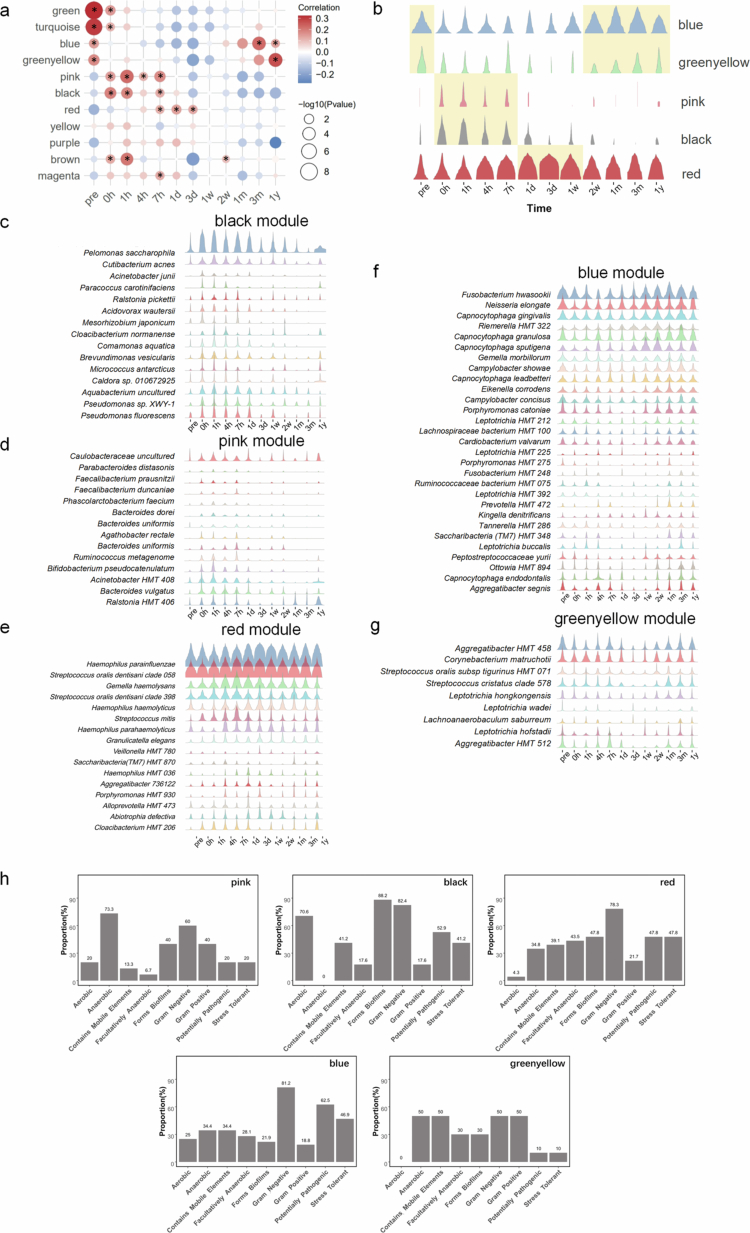
Reconstruction stages and covariant species modules of subgingival microbiota. (a) Heatmap of module–time associations. The abscissa represents the bacterial module, while the ordinate represents the time point. The intersection of dots on the abscissa and ordinate indicates their correlation, with the magnitude of correlation indicated by the color and shape legend. *: The positive correlation is statistically significant (*p* < 0.05). (b) Temporal variation in the relative abundance of covariant species modules during early (pink and black), middle (red), and late (blue and green–yellow) periods. The species contained in modules (c) black, (d) pink, (e) red, (f) blue, (g) green-yellow, and their relative abundance variation trends are highlighted. (h) Microbiome phenotype prediction by BugBase, including characteristics such as aerobic, anaerobic, facultatively anaerobic, contains mobile elements, biofilm forming, gram-positive, gram-negative, pathogenic potential, and stress tolerance.

To elucidate the intrinsic drivers underlying microbial community restructuring, we investigated the relationship between covariant species modules and relative abundance shifts. Our analysis revealed that subgingival microbiota reorganization was mediated through coordinated changes in ecologically linked species groups, with significant positive correlations observed among member taxa within each functional module. Subgingival microbiome species were classified into 12 covariant species modules using weighted correlation network analysis (WGCNA), and these were further associated with specific time points (Suplementary Figure S2). The reconstruction process of subgingival microbiome was divided into three stages: the early stage (including hours 0, 1, 4 and 7), the middle stage (including days 1, 3, and week 1), and the late stage (including week 2, months 1, 3, and year 1). The results indicated that the Pink and Black modules were positively related to the early stage, with the relative abundance of bacteria in these modules increasing initially and then decreasing. The Red module was positively correlated with the middle stage of reconstruction, reaching peak abundance during this phase. The blue and green–yellow modules were positively correlated with both the late reconstruction period and the pre-operation period. The microbiome abundance in these modules decreased post-operation and recovered to a state similar to the pre-operation period during the late stage (Figure 3a,b).

The species within each module and their trends in relative abundance are presented in Figure 3c–g. The early representative Black module contained 15 species, including *Pelomonas saccharophila*, *Cutibacterium acnes* and *Acinetobacter junii*. The early Pink module contained 14 low-abundance species, such as *Acinetobacter HMT 408*, *Ralstonia HMT 406,* and *Caulobacteraceae uncultured*. The red module included 16 species, with *Haemophilus parainfluenzae* having the highest relative abundance, alongside other high-abundance species such as *Streptococcus oralis 058*, *Streptococcus oralis 398*, *Gemella haemolysans,* and *Haemophilus haemolyticus*. The late blue and green–yellow modules contained 29 and 9 species, respectively, with *Fusobacterium hwasookii*, *Neisseria elongate,* and several *Capnocytophaga* species (such as *Capnocytophaga gingivalis*, *Capnocytophaga granulosa*, *Capnocytophaga sputigena,* and *Capnocytophaga leadbetteri*) being high-abundance species in the blue module. Similarly, *Aggregatibacter HMT 458* and *Corynebacterium matruchotii* were predominant in the green–yellow module. The phenotypic characteristics of representative modules were further predicted using Bugbase. Figure 3h indicated that gram-negative bacteria dominated all five modules, with anaerobic bacteria prevailing in the early pink module, while aerobic bacteria dominated the black module, which also exhibited exceptional biofilm-forming capability. Notably, bacterial taxa within the late-stage blue modules demonstrated the highest pathogenic potential.

### The reconstruction process is dictated by bacterial modules’ functional potential

**Figure 4.**
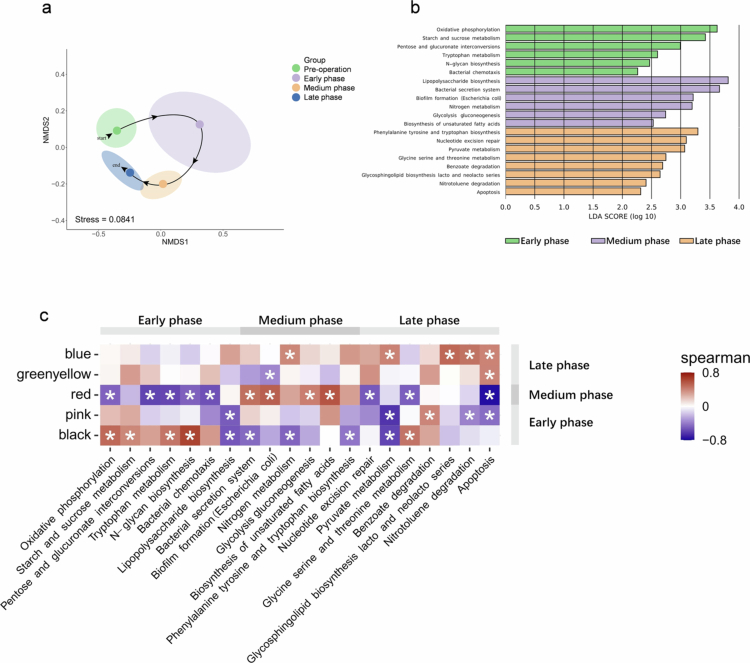
Functional characteristics of subgingival microbiota reconstruction. (a) Non-metric multidimensional scaling (NMDS) of functional items in preoperative, early, medium, and late phases. (b) Linear discriminant analysis effect size (LEfSe, *α* = 0.05, log_10_ LDA score > 2.0) identified functional biomarkers in early, medium, and late phases. (c) Spearman correlations between the average relative abundance of characteristic species modules (identified by full-length 16S rRNA gene sequencing) and the relative expression of functional entries (identified by metagenomic sequencing).

To determine the temporal dynamics of microbiota functional capacity profiles, we sequenced the metagenome of 30 subgingival plaque samples selected via micro-PITA from early, middle, and late stages. The initial investigation into the temporal profiles of the subgingival microbiome provided fundamental insights. Metagenomic analysis indicated that species variation in pre-operation, early, middle, and late phases paralleled the ‘regression after offset’ trend observed in full-length 16S rRNA gene sequencing results (Suplementary Figure S3). Additionally, the evolution of subgingival microbiome functional capacity followed similar trajectories (Figure 4a). We then applied linear discriminant analysis (LDA) effect size (LEfSe) to identify stage-specific enrichment of functional capacity (Figure 4b). Early phase functional profiles, including oxidative phosphorylation, starch and sucrose metabolism, pentose and glucuronate interconversions, tryptophan metabolism, *N*-Glycan biosynthesis, and bacterial chemotaxis, were enriched. During the middle phase, predominant functional profiles included lipopolysaccharide biosynthesis, bacterial secretion, biofilm formation, nitrogen metabolism, glycolysis gluconeogenesis, and biosynthesis of unsaturated fatty acids. In the late phase, enriched functional capacities were phenylalanine, tyrosine and tryptophan biosynthesis, nucleotide excision repair, pyruvate metabolism, glycine, serine and threonine metabolism, benzoate degradation, glycosphingolipid biosynthesis of the lacto and neolacto series, nitrotoluene degradation, and apoptosis. This suggests that the reconstruction process of subgingival microbiome was potentially initiated by energy metabolism in the early stage, expanded through biofilm formation and LPS biosynthesis in the middle stage, and constrained by apoptosis, while also fostering abundant and complex amino acid metabolism. Subsequently, we performed Spearman correlation analysis on paired samples to examine the associations between the average relative abundance of characteristic species modules (obtained by full-length 16S rRNA gene sequencing) and the relative expression of functional entries (obtained by metagenomic sequencing) (Figure 4c). The results demonstrated a positive correlation between the black module, which includes *P. saccharophila*, and functional capacities such as oxidative phosphorylation, starch and sucrose metabolism, particularly *N*-Glycan biosynthesis. The mid-red module, containing *S. oralis 058* and *S. oralis 398*, *G. haemolysans,* and *H. haemolyticus,* was dominant in biofilm formation and the biosynthesis of unsaturated fatty acids. In the late stage, the Blue module, featuring *F. hwasookii*, *N. elongate*, and various *Capnocytophaga* species, predominantly exhibited positive correlations with nitrotoluene degradation and apoptosis.

### Internal driving forces among subgingival microbiota reconstruction

**Figure 5.**
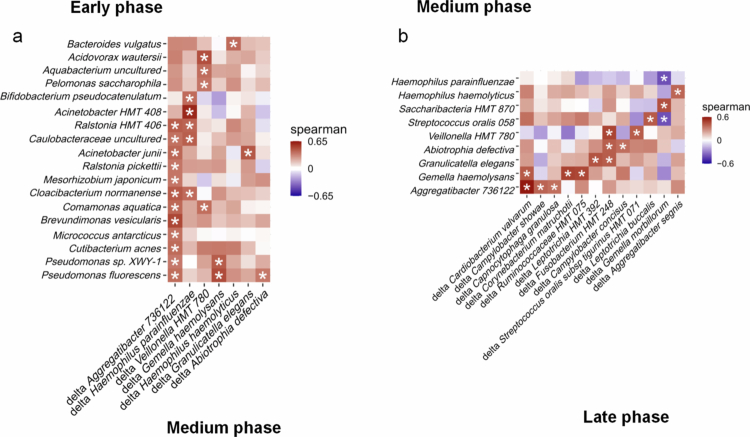
Internal driving forces of subgingival microbiota reconstruction. (a) Heatmap of delta correlation analysis between early and medium characteristic species. (b) Heatmap of delta correlation analysis between medium and late characteristic species. *: The correlation is statistically significant.

In this study, we employed delta correlation analysis to construct matrices between the relative abundance of characteristic species at each stage (such as the early stage) and the abundance variables at the later stage (such as the medium stage) for Spearman correlation analysis. We defined pairs with time-oriented positive correlations as potential driving relationships, implying that a higher relative abundance of bacteria A would promote the growth of bacteria B in a subsequent stage. Delta correlation analysis revealed a significant positive correlation between the increased relative abundance in the medium red module and the abundance in the pink and black modules during the early stage (Figure 5a). Specifically, 12 species, dominated by *Ralstonia. HMT 406, Ralstonia pickettii* were positively associated with an increased abundance of *Aggregatibacter 736122*. 6 species, for instance, *Acinetobacter HMT 408*, *Ralstonia. HMT 406*, were positively associated with an increased abundance of various *Haemophilus species,* including *H. parainfluenzae,* and *H. haemolyticus*. The expansion of *Veillonella HMT 780* was driven by 4 early-phase strains, including *Acidovorax wautersii*, *Aquabacterium uncultured*, *P. saccharophila* and *Comamonas aquatica.* Additionally, the expansion of *G. haemolysans* was driven by *Pseudomonas sp. XWY-1* and *P. fluorescens*. Furthermore, *A. junii* promoted the proliferation of *Granulicatella elegans*, and the expansion of *Abiotrophia defective* was driven by *P. fluorescens*.

Regarding delta correlation analysis between bacteria in the medium Red module and those in the late Blue/Greenyellow module (Figure 5b), the proliferation of three species, including *Cardiobacterium valvarum*, *Campylobacter showae,* and *C. granulosa,* was promoted by *Aggregatibacter 736122*. Similarly, *G. haemolysans* was positively associated with an increased abundance of *C. valvarum, C. matruchotii,* and *Ruminococcaceae HMT 392*. Moreover, *G. elegans, A. defective,* and *Veillonella HMT 780* promoted the expansion of two late-stage species, respectively *Leptotrichia HMT 392* and *Fusobacterium HMT 248*, *Fusobacterium HMT 248* and *Campylobacter concisus*, *Fusobacterium HMT 248* and *Streptococcus oralis subsp tigurinus HMT 071*. The expansion of *Leptotrichia buccalis, Gemella morbillorum,* and *Aggregatibacter segnis* was driven respectively by *S. oralis 058*, *Saccharibacteria HMT 870,* and *H. haemolyticus*. The driving forces between subgingival species across different periods were systematically illustrated in Figure 5a,b.

## Discussion

Ecological characteristics such as stability, resistance, and resilience are crucial for host health. Perturbations can cause a stable microbial ecosystem to be briefly pushed into an unstable state [24–26]. Subsequently, this ecosystem may return to its initial state or transition to another stable state, either healthy or unhealthy [27]. The oral cavity experiences frequent disturbances from oral behaviors such as eating, tooth brushing, gargling, and smoking [28]. The stability and variability of oral microbes have been extensively investigated [25,29,30]. In this study, scaling was employed as a perturbation to the subgingival microbiome. Compared to the initial state, the subgingival microbiota exhibited its most heterogeneous state on day 3 after the disturbance and subsequently recovered over the following week to a year, mirroring findings from previous studies [31]. Li et al. observed that the microbial community in various periodontal initiation states (healthy periodontal, gingivitis, and periodontitis) returned to its original state at different rates following plaque removal [22]. Functional redundancy, conferred by microbial diversity, is considered a significant factor in the resilience of microbial communities, mediating recoverable changes in microbial structure and function within a threshold range through a ‘compensatory action’ [32,33].

Furthermore, we conducted an in-depth analysis from the perspective of microbiome structure and function. The 1-year recovery time was initially divided into early, middle, and late periods through clustering, followed by the identification of covariant species modules for each period using WGCNA. Notably, we identified a group of low-abundance characteristic microorganisms during the early stages, including species such as *P. saccharophila*, *Micrococcus antarcticus*, *Mesorhizobium japonicum*, and *C. aquatica*. These taxa, which are not typically associated with the oral microbiota, emerged within hours after ultrasonic scaling. We hypothesize that these microbes represent transient species originating from external environments. The oral cavity, as an open ecosystem, undergoes continuous microbial exchange with the external environment via breathing, dietary intake, and fluid consumption. Consequently, microorganisms derived from environmental sources, food, or other body sites (such as the skin or intestinal tract) may transiently colonize the oral cavity, forming a temporary microbial assemblage. Although these bacteria are unlikely to establish permanent colonization, they can be captured during sampling and thus contribute to the temporal variability of the oral microbiome.

High-abundance species such as *S. oralis 058*, *S. oralis 398*, *G. haemolysans,* and *H. haemolyticus* were characteristic microorganisms of the middle period. In previous studies, *Streptococci*, particularly *Streptococcus mitis* and *S. oralis*, along with *Neisseria*, *Rothia*, and *Gemella,* have been identified as early colonizers in supragingival plaque formation. The delayed emergence of *Streptococcus* and *Gemella* in subgingival plaque could be attributed to their location deep within the gingival sulcus, necessitating a longer period for bacterial migration from external sources, saliva, and supragingival microbiome. Interestingly, late-stage microbes, including *Fusobacterium nucleatum*, *Leptothrix*, and *Capnocytophaga*, may play roles in the pathogenesis of perodontal diseases [34–36]. It has been suggested that plaque accumulation acts as a bridge between periodontal health and disease, with mature plaque potentially serving as a reservoir for subsequent colonization by periodontal pathogens [37]. Metagenomic sequencing revealed functional capacity shifts during the reconstruction of subgingival microbiome, initiated by energy metabolism, expanded by biofilm formation and LPS biosynthesis, and constrained by apoptosis, while facilitating abundant and complex amino acid metabolism. Compared with healthy subjects, the subgingival microbiome of periodontitis patients exhibited up-regulated genes encoding virulence factors, amino acid metabolism, glycosaminoglycan degradation, and pyridine degradation, and down-regulated genes encoding carbohydrate metabolism [38–40]. Like shifts in microbial composition, these functional capacity changes contribute to the establishment of a ‘periodontitis-oriented state’. It is worth noting that our metagenomic sequencing identifies the presence and abundance of genes encoding specific metabolic functions within the microbial community, but it does not directly measure their transcriptional activity. Therefore, our analysis reveals time-dependent and context-dependent shifts in the functional capacity of the community. Validation of the actual expression and activity of these pathways would require complementary metatranscriptomic and metabolomic analyses in future work.

What dynamics drive the recovery process of a disturbed community? We initially approached this issue macroscopically from an ecological perspective. Our analysis revealed that the reconstruction of subgingival microbiome primarily involves homogeneous selection and dispersal limitation. We suggest that communities tend to establish in more similar patterns under consistent biological/abiotic conditions, while the movement and establishment of taxa in new locations are constrained. Our findings are consistent with those of Chen et al. who noted in their assembly analysis of subgingival microbiota in healthy individuals that dispersal limitation and homogeneous selection are predominant, indicating restricted transmission of subgingival microbiome between individuals and influence by local environmental factors of the gingival sulcus. However, our results show a stronger influence of homogeneous selection over dispersal limitation, which emphasizes dispersal limitation. This difference could be due to variations in sample characteristics and nuances in algorithm implementation [41].

The temporal dynamics of microbiomes are shaped by three primary factors: (i) biotic and abiotic history, (ii) intrinsic community dynamics, and (iii) external perturbations. Within the gingival crevice, local microenvironmental conditions – such as oxygen tension, pH, and nutrient availability – serve as critical determinants of subgingival microbial community restructuring. Meanwhile, intrinsic ecological processes, including evolutionary adaptation, interspecies interactions, and feedback mechanisms arising from microbial niche modification, further drive temporal variability [42,43]. Microbial interactions play a pivotal role in community function and evolutionary trajectories [42,44]. While co-occurrence network analysis has become a cornerstone of microbial ecology [45,46], this approach fails to capture directional relationships or causal mechanisms [47]. Recent advances in dynamical systems modeling have transcended descriptive community profiling, enabling mechanistic insights into microbial assembly rules [48]. Among these, the Generalized Lotka-Volterra (GLV) framework has emerged as a powerful tool for modeling multi-species ecological dynamics in host-associated microbiomes [49,50].

To elucidate temporal microbial interactions, we employed delta correlation analysis to identify directional relationships between bacterial modules across successive colonization periods. This analysis revealed how abundance variations of taxa influenced subsequent microbial succession rates. Although our study observed that several mid-stage taxa were influenced by early colonizing and transient microorganisms, whether these early microbial arrivals exert biologically significant driving effects on the highly abundant mid-stage oral species remains to be further validated. We therefore focused specifically on interactions among mid- and late-stage module bacteria. Our results demonstrate cooperative relationships among several core oral residents, including *Veillonella HMT 780*, *A. defectiva*, *G. elegans*, and *Fusobacterium HMT 248*. In addition, a mutually promotive interaction was observed between *Veillonella HMT 780* and *Streptococcus oralis subsp tigurinus HMT 071*. These findings align with previously reported interactions. For example, *Veillonella* and *F. nucleatum* are known to coaggregate physically [51]. The catalase produced by *Veillonella* can protect *F. nucleatum* from oxidative stress [52]. Furthermore, *Veillonella* enhances lysine availability, thereby promoting cadaverine production by *F. nucleatum* [53]. Similarly, coaggregation has been documented between *G. elegans* and *F. nucleatum* [54], as well as between *Veillonella* and various streptococci [55]. Microbial interactions in biofilms occur through multiple mechanisms, including physical adhesion, quorum sensing, and metabolic cross-feeding [56,57]. Representative examples include *S. mitis* supplying NAD to *H. parainfluenzae*, and *Actinomyces oris* generating lactic acid that supports the growth of *Veillonella parvula*. The dynamically evolving network of interbacterial relationships observed within *in situ* dental plaque provides novel insights into the mechanisms underlying microbial interactions.

This study has several limitations that should be considered. Firstly, subgingival plaque samples from different sites were pooled for analysis. While this approach was necessary to obtain sufficient microbial biomass for 16S rRNA gene sequencing and metagenomic analysis, it inherently averages the microbial community data. Thus, our findings reflect a generalized microbiota profile and could miss site-specific variations influenced by local microenvironments. Future studies using single-site sampling or spatial techniques would help clarify the biogeography of the subgingival microbiome. Then, to enhance the capture of oral microbial diversity, we employed a sequential taxonomic annotation approach using the eHOMD, SALIVA, and GenBank databases. This strategy improved species-level identification but also led to the detection of relatively uncommon oral bacteria or taxa often associated with environmental sources – particularly during the early restructuring phase, when the native oral microbiota was suppressed and not yet dominant. The presence of bacterial taxa commonly linked to non-oral environments may reflect transient microorganisms introduced via diet or respiration. The detection of such microorganisms, while indicative of the authentic compositional features under real-world sampling conditions, necessitates heightened critical scrutiny regarding the biological relevance of putative keystone taxa and their inferred network interactions.

## Conclusions

In summary, our research clarifies the species and functional characteristics involved in the reconstruction process of the disturbed subgingival microbiome. It maps time-directed networks among stage-specific bacteria, providing a crucial theoretical framework for predictable targeted microbiome regulation.

## Supplementary Material

Supplementary material **Supplementary Table 1.** Basic characteristics of subjects at baseline. **Supplementary Table 2.** Clinical information of subjects at 3 month and 1 year. **Supplementary Figure S1.** The composition and structure of the subgingival microbiome before and after surgery at 3 months and 1 year. **Supplementary Figure S2.** Weighted correlation network analysis (WGCNA). **Supplementary Figure S3.** Reconfiguration of subgingival microbiota after disturbance revealed by metagenomic sequencing.

## Data Availability

The sequencing data have been deposited in the Sequence Read Archive under accession BioProject PRJNA1158003.
