# Supplementary material for: Meta-omics reveals subgingival plaque reconstruction dynamics
Source: J Oral Microbiol. 2025 Oct 13;17(1):2569528. doi: 10.1080/20002297.2025.2569528 (PMC12519584; doi:10.1080/20002297.2025.2569528)
Supplement: Supplementary material — Supplementary Table 1. Basic characteristics of subjects at baseline. Supplementary Table 2. Clinical information of subjects at 3 month and 1 year. Supplementary Figure S1. The composition and structure of the subgingival microbiome before and after surgery at 3 months and 1 year. Supplementary Figure S2. Weighted correlation network analysis (WGCNA). Supplementary Figure S3. Reconfiguration of subgingival microbiota after disturbance revealed by metagenomic sequencing. [file ZJOM_A_2569528_SM4783.docx]

**Supplementary Table 1 Basic characteristics of subjects at baseline**

| Environmental factors | Statistics (N, Mean±SD) |
| --- | --- |
| Gender (F/M) | 20/10 |
| Age | 22.10±1.99 |
| BOP (%) | 4.35±0.03 |
| PD (mm) | 1.98±0.06 |

**Supplementary Table 2 Clinical information of subjects at 3 month and 1 year**

|  | Statistics (Mean±SD) | |
| --- | --- | --- |
| Clinical indexes | 3m (N=29) | 1y (N=22) |
| BOP (%) | 4.44±0.03 | 4.17±0.03 |
| PD (mm) | 1.49±0.08 | 1.49±0.11 |

**Supplementary Figure S1 The composition and structure of the subgingival microbiome before and after surgery at 3 months and 1 year.**

**(a)** Principal coordinates analysis (PCoA) of subgingival bacteria before and after surgery at 3 months and 1 year. (**b)** Community structures via the relative abundance of the top 30 species of subgingival bacteria before and after surgery at 3 months and 1 year.

**Supplementary Figure S2 Weighted correlation network analysis (WGCNA).**

1. Calculation and selection of optimal soft-thresholding powers. The power=12 was used in this analysis. **(b)** Module clustering tree diagram. The establishment of a weighted co-expression network model was based on the selected power value, resulting in the division of all species into 11 modules (except the gray module).


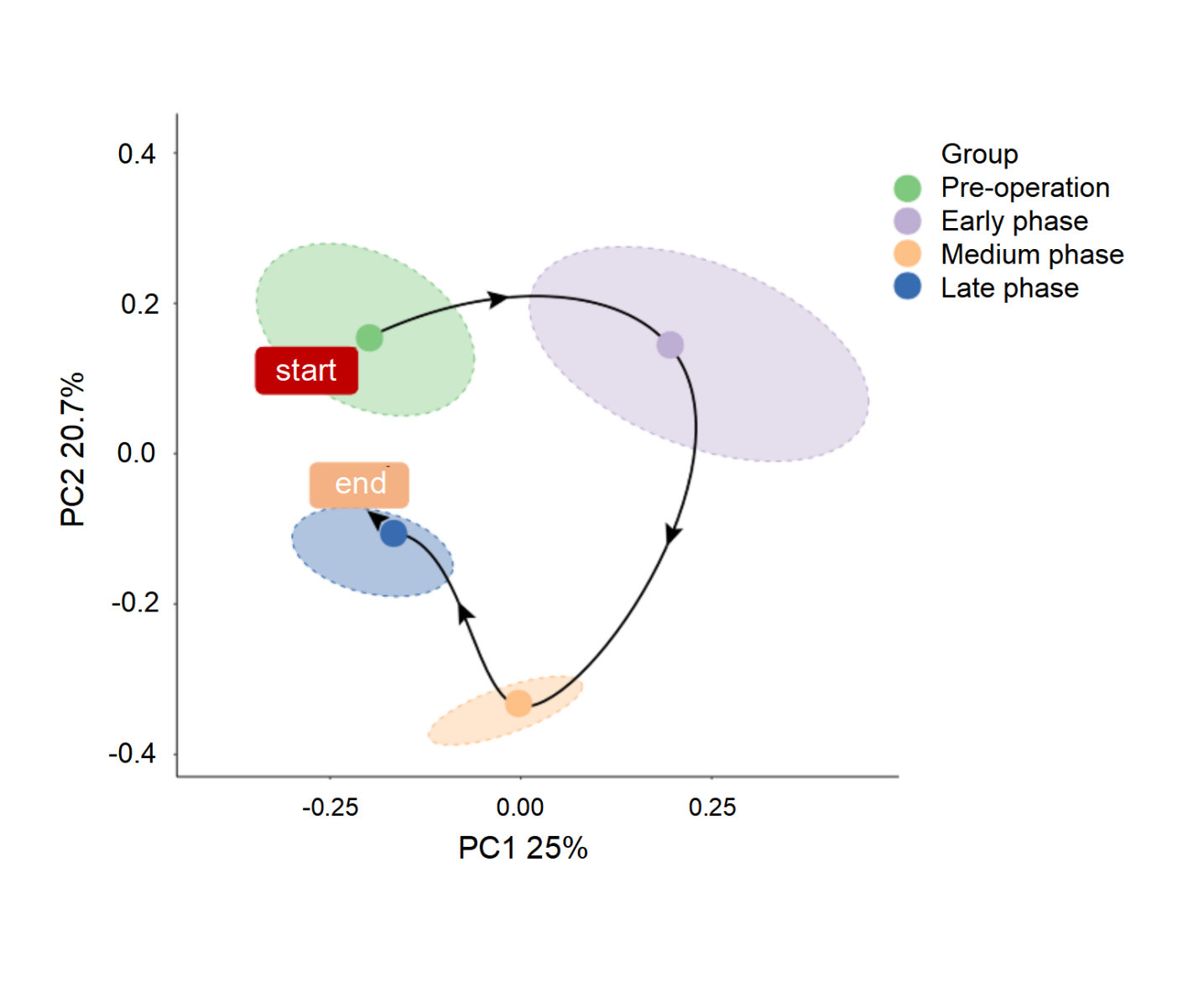


**Supplementary Figure S3 Reconfiguration of subgingival microbiota after disturbance revealed by metagenomic sequencing**

Non-Metric Multidimensional Scaling (NMDS) of species-level microbial taxa composition.
